# Superbugs in the supermarket? Assessing the rate of contamination with third-generation cephalosporin-resistant gram-negative bacteria in fresh Australian pork and chicken

**DOI:** 10.1186/s13756-018-0322-4

**Published:** 2018-02-23

**Authors:** Jade E. McLellan, Ashleigh J. Pitcher, Susan A. Ballard, Elizabeth A. Grabsch, Jan M. Bell, Mary Barton, M. Lindsay Grayson

**Affiliations:** 1 Department of Medicine, Austin Health, University of Melbourne, Melbourne, VIC Australia; 2 Infectious Diseases & Microbiology Departments, Austin Health, Melbourne, VIC Australia; 3 Infectious Diseases and Microbiology, SA Pathology, Adelaide, South Australia Australia; 4 School of Pharmacy and Medical Sciences, University of South Australia, Adelaide, South Australia Australia; 5 Department of Epidemiology and Preventive Medicine, Monash University, Melbourne, VIC Australia

**Keywords:** Infection, Antibiotic resistance, Foodborne

## Abstract

**Background:**

Antibiotic misuse in food-producing animals is potentially associated with human acquisition of multidrug-resistant (MDR; resistance to ≥ 3 drug classes) bacteria via the food chain. We aimed to determine if MDR Gram-negative (GNB) organisms are present in fresh Australian chicken and pork products.

**Methods:**

We sampled raw, chicken drumsticks (CD) and pork ribs (PR) from 30 local supermarkets/butchers across Melbourne on two occasions. Specimens were sub-cultured onto selective media for third-generation cephalosporin-resistant (3GCR) GNBs, with species identification and antibiotic susceptibility determined for all unique colonies. Isolates were assessed by PCR for SHV, TEM, CTX-M, AmpC and carbapenemase genes (encoding IMP, VIM, KPC, OXA-48, NDM).

**Results:**

From 120 specimens (60 CD, 60 PR), 112 (93%) grew a 3GCR-GNB (*n* = 164 isolates; 86 CD, 78 PR); common species were *Acinetobacter baumannii* (37%), *Pseudomonas aeruginosa* (13%) and *Serratia fonticola* (12%), but only one *E. coli* isolate. Fifty-nine (36%) had evidence of 3GCR alone, 93/163 (57%) displayed 3GCR plus resistance to one additional antibiotic class, and 9/163 (6%) were 3GCR plus resistance to two additional classes. Of 158 DNA specimens, all were negative for ESBL/carbapenemase genes, except 23 (15%) which were positive for AmpC, with 22/23 considered to be inherently chromosomal, but the sole *E. coli* isolate contained a plasmid-mediated CMY-2 AmpC.

**Conclusions:**

We found low rates of MDR-GNBs in Australian chicken and pork meat, but potential 3GCR-GNBs are common (93% specimens). Testing programs that only assess for *E. coli* are likely to severely underestimate the diversity of 3GCR organisms in fresh meat.

**Electronic supplementary material:**

The online version of this article (10.1186/s13756-018-0322-4) contains supplementary material, which is available to authorized users.

## Background

The emergence of multi-drug resistant (MDR) bacteria is a major health problem that has been likened in its global future impact on human health to that of terrorism [1, 2]. Widespread inappropriate use of antimicrobials in food production (especially meat/seafood, some fruit) has been linked to environmental contamination with MDR pathogens and outbreaks of MDR infections in humans, but direct cause-and-effect has often been difficult to confirm, despite the strength of the observed associations [3–8]. Most food testing programs for antimicrobial resistance (AMR) have focused on specific organisms (e.g. *E. coli*, Salmonella *spp.*, Listeria *spp.*), assuming direct food-to-human pathogen transfer, rather than considering resistant gene transfer between bacterial species [6, 8, 9]. Furthermore, the optimum site of specimen collection (e.g. on-farm animal, manure, abattoir, point-of-sale supermarket products) has been debated [9–13]. Although Australia has reasonably strict regulations regarding antimicrobial use in agriculture [13, 14], use of some agents for prophylaxis and treatment (e.g. trimethoprim-sulfamethoxazole, some beta-lactams and macrolides) is common in some food sectors [2, 13, 15, 16], such that this may have some implications for acquisition by consumers of multi-resistant pathogens via food consumption [6, 7].

Hence, we aimed to assess the rates of contamination with potential extended-spectrum beta-lactamase (ESBL)-producing Gram-negative organisms (without restricting to specific species) in Australian-produced chicken and pork meat. To best identify any potential risk to the consumer and to be certain that the meat was produced in Australia, we purchased chicken drumsticks and pork ribs at local fresh food outlets, since national legislation requires that bone-containing meat products must be Australian-produced (by conventional or organic production), whereas de-boned meats (e.g. bacon) can be imported into Australia [17].

## Methods

### Study design

This was a prospective cross-sectional survey undertaken during a four-month period from March to June 2014 in the eastern suburbs of Melbourne, Australia. We identified ten regions within the medical catchment area of the Austin Hospital and sampled from 2 to 4 retailers within each region (see Additional file 1: Figure S1 for locations). We tested raw, skin-covered chicken drumsticks (CD) and pork spare ribs (PR), each weighing approximately 150 g, from a total of 30 meat retailers (26 supermarkets, 4 butchers shops). Samples were purchased from each site on two occasions (approximately one month between each sample). Each sample from a supermarket was derived from a pre-packaged container with multiple CD or PR specimens, while each sample from a butcher was selected for purchase individually and was not pre-packaged.

### Specimen handling, culture and susceptibility

Similar to methods previously described [18, 19], each specimen was placed individually into separate zip lock bags (22 × 22 cm, Hercules, Australia), to which 100 mL of buffered peptone water (Thermofisher Scientific, Australia) was added and the specimen was massaged manually for 2 min. Of the subsequent rinsate, 50 mL was added in a sterile manner to 50 mL of double-strength tryptone soya broth (TSB; Thermofisher Scientific, Australia) which was incubated for 24 h at 37 °C. From this broth, 100 μL was inoculated into 10 mL of TSB containing ceftriaxone (0.25 mg/L) and vancomycin (8 mg/L), and incubated (37 °C, 24 h) before 10 μL was inoculated and spread onto ChromID ESBL agar (BioMérieux, France) and incubated for 48 h at 37 °C [18, 19]. All unique colonial morphologies on this selective medium were purity-plated onto Columbia horse blood agar/MacConkey agar (HBA/MAC; Thermofisher Scientific, Australia) and then subcultured onto Columbia HBA (Thermo Scientific, Australia) and incubated (37 °C, 24 h) before being identified using MALDI-TOF MS (BioMérieux, France) and tested for antibiotic susceptibility by Vitek2® (BioMérieux, France) using CLSI clinical breakpoint criteria. For those species and antibiotics where there were no defined criteria (e.g. *Pseudomonas* spp., *Stenotrophomonas* spp.), the Vitek-derived MIC value was compared to the relevant EUCAST distribution to categorise (for the purpose of this study) the presence of resistance (either intrinsic or acquired) [20–23]. If MALDI-TOF MS was unable to confidently identify (< 90% match) the organism after three attempts, Vitek2® was used for identification.

Isolates which grew on ChromID ESBL agar and displayed phenotypic resistance by Vitek2 to third-generation cephalosporins (ceftriaxone; 3GCR) were considered to be potential ESBL-producers or intrinsically 3GCR [24–26] and were classified according to the number of antibiotic classes to which they were resistant – including third-generation cephalosporins, carbapenems (meropenem), aminoglycosides (gentamicin), fluoroquinolones (ciprofloxacin) and anti-folates (trimethoprim-sulfamethoxazole). Similar to previously, multi-drug-resistance (MDR) was defined as resistance to ≥3 classes of antibiotics [27].

### Molecular assessment for beta-lactamase genes

DNA was extracted from all potential ESBL-producing isolates using previously described methods (DNeasy Blood and Tissue kit, Qiagen, USA), then screened for the presence of the bla_TEM_, and bla_SHV_ genes using a real-time polymerase chain reaction (PCR) platform (LC-480) and published primers [28, 29]. A multiplex real-time TaqMan PCR was used to detect CTX-M-type genes (groups 1, 2, 9, 8, 25) [30]. Strains were probed for plasmid-borne AmpC enzymes using the method described by Pérez-Pérez and Hanson (including blaACC-like, blaDHA-like, blaCIT/CMY-like, blaMOX-like, blaFOX-like, blaMIR/ACT-like; [31]) and subjected to molecular tests for MBL (bla_VIM_, bla_IMP_, and bla_NDM_), bla_KPC_, and blaOXA-48-like genes using real-time PCR [32, 33]. Isolates suspected of containing transferable ESBL or MBL genes underwent whole genome sequencing whereby unique dual indexed libraries were prepared from genomic DNA using the Nextera XT DNA sample preparation kit (Illumina). Libraries were sequenced on the Illumina NextSeq 500 with 150-cycle paired end chemistry as described by the manufacturer’s protocols and sequences were accessed for known resistance genes using KmerResistance 2.2 [34].

### Data analysis and statistics

The rates of contamination with potential ESBL-producing Gram-negative organisms were assessed according to specimen type (CD, PR), the geographic site of specimen purchase and the type of meat outlet (supermarket vs butcher). Similar rates were reported for PCR-confirmed ESBL isolates and those where the ESBL was likely to be plasmid-mediated. Comparisons between rates for CD and PR were undertaken using Chi-square.

## Results

Of a total of 120 meat specimens (60 CD, mean ± SD weight: 155.4 ± 26.5 [range 78.5–223.9] grams; 60 PR, 160.5 ± 48.9 [range: 91.5–355.1] grams) that were assessed from 30 retailers (see locations in Additional file 1: Figure S1), 112 (56 CD, 93%; 56 PR; 93%) were contaminated with a total of 164 (86 CD; 78 PR) 3GCR (i.e. potential ESBL-producing) isolates (Table 1). Among these isolates, 59 (36%; 26 CD, 33 PR) displayed phenotypic evidence of 3GCR alone, 96 (59%; 54 CD, 42 PR) were 3GCR plus were also resistant to either anti-folates, aminoglycosides or carbapenems and 9 isolates (5.5%; 6 CD, 3 PR; 9 specimens; 5 *Pseudomonas aeruginosa*, 2 *Pseudomonas* spp., 1 *Bordetella trematum*, 1 *Chryseobacterium gleum*) were MDR with evidence of being 3GCR plus resistance to two other antibiotic classes. Resistance to anti-folates was most common (*n* = 91 [55%] isolates, 49 CD, 42 PR, Table 1; 82 [68.3%] specimens). The four most common 3GCR species identified were *Acinetobacter baumannii* complex (*n* = 59), *Pseudomonas aeruginosa* (*n* = 22), *Serratia fonticola* (*n* = 19) and *Hafnia alvei* (*n* = 15). Only one *E. coli* isolate was identified – this was in a CD specimen.

**Table 1:** Summary of isolates grown from fresh retail chicken and pork

Isolate		Chicken							Pork							
	Overall total *n* = 164	Total *n* = 86	3GCR alone	3GCR + one class		3GCR + two classes			Total *n* = 78	3GCR alone	3GCR + one class			3GCR + two classes		
				AF	Mero	AF + Mero	AF + FQ	Mero+AMG			AF	Mero	AMG	AF + Mero	AF + FQ	Mero + AMG
*Acinetobacter baumannii* complex	59	34	–	34	–	–			25	–	25	–	–	–		
*Acinetobacter ursingii*	2	–							2	–	2	–	–	–		
*Aeromonas sobria*	1^f^	–							1	–	–	1	–	–		
*Bordetella trematum*	1	–							1	–	–	–	–	–	1	–
*Chryseobacterium gleum*	1	–							1	–	–	–	–	–	–	1
*Citrobacter braakii*	7	–							7^b^	7^b^	–	–	–	–		
*Citrobacter freundii*	6^e^	–							6	6	–	–	–	–		
*Citrobacter youngae*	1^e^	1	1	–	–	–			–							
*Enterobacter cloacae* complex	9^g^	1	1	–	–	–			8	8	–	–	–	–		
*Escherichia coli*	1^a^	1^a^	1^a^	–	–	–			–							
*Hafnia alvei*	15^c^	6	6	–	–	–			9	9	–	–	–	–		
*Pseudomonas aeruginosa*	22	11	–	7	–	4	–	–	11	–	10	–	–	1	–	–
*Pseudomonas alcaligenes*	1	1	–	–	1	–			–							
*Pseudomonas oleovorans*	3	1	1	–	–	–			2	–	2	–	–	–		
*Pseudomonas putida*	11	10	1	1	8	–			1	–	1	–	–	–		
*Pseudomonas* spp.	3	3	–	–	1	2	–	–	–							
*Serratia fonticola*	19^d^	16	15	1	–	–			3	3	–	–	–	–		
*Stenotrophomonas maltophilia*	1	–							1	–	–	–	1	–		
*Yokenella regensburgei*	1	1	–	–	1	–			–	–	–	–	–	–		

*3GCR* Third-generation cephalosporin resistance, *AF* Anti-folates, *FQ* Fluoroquinolones, *Mero* Meropenem, *AMG* Aminoglycosides

^a^*E. coli* was an ST 349 isolate and contained a plasmid-mediated CMY-2 AmpC ESBL

^b^Includes 4× *C. braakii/freundii* and 1× *C. braakii/werkmanii* isolates

^c^10 *H. alvei* isolates contained ACC AmpC ESBLs - 4 were from chicken and 6 from pork

^d^Two *S. fonticola* isolates contained ACC AmpC ESBLs – 1 was from chicken and 1 from pork

^e^All isolates contained CMY-like AmpC ESBL

^f^Isolate contained a FOX AmpC ESBL

^g^Two isolates contained an EBC AmpC ESBL – both from pork

Among the 164 isolates, 158 had DNA available for PCR analysis. Beta-lactamase genes were identified in 23 (15%) isolates (7CD, 14PR [2 PR each had two isolates], *p* = 0.15; 17.5% specimens). All were AmpC, with 22/23 considered to be inherently chromosomally located (ACC, *n* = 12 [*H. alvei*, 10; *S. fonticola*, 2]; CMY-like *n* = 7 [*C. freundii*, 6; *C. youngae/freundii*, 1]; FOX, *n* = 1 [*A. sobria*]; MIR-like/ACT-like, *n* = 2; [*E. cloacae* complex]), while the sole *E. coli* isolate contained a CMY-like AmpC gene that was likely to be plasmid-mediated and was subsequently shown on whole genome sequencing to be a CMY-2 (see Table 1). All DNA samples were PCR-negative for other ESBL genes (including SHV, TEM, CTX-M) and all carbapenemase encoding gene families (including IMP, VIM, KPC, OXA-48-like and NDM).

Among the 30 food outlets, there were four supermarket chains (two large [*n* = 10 and 11 stores sampled]; two smaller [*n* = 2 and 3 stores] and 4 separate (unlinked) butcher shops. Overall, there were no differences in rates of contamination between supermarkets and unlinked butchers shops. All supermarkets and butcher shops had at least one CD or PR specimen that grew a potential ESBL-producing isolate, at some time. Only 8 specimens were culture-negative (4 CD, 4 PR; one supermarket site had both its PR specimens culture-negative). The numbers of 3GCR isolates per specimen were as follows: single isolate in 63 specimens; two isolates in 44 specimens; 3 isolates in 3 specimens, and one specimen contained 4 potential ESBL-producing isolates. Interestingly, it was this latter specimen (which was collected from a butcher’s shop) that grew the CMY-2-containing *E. coli*, along with an *A. baumannii*, *S. fonticola* and an *E. cloacae* complex isolate – although none of these latter 3 isolates contained any definable ESBL genes (Table 1).

## Discussion

This study of Australian chicken and pork is notable for a number of reasons. Firstly, we assessed for a broad range of Gram-negative organisms, not simply the traditional species of *E. coli* or Salmonella *spp.* [6, 8–10]. Taking this approach, we identified that 93% of specimens appeared to be contaminated with a wide variety of 3GCR species, including particularly *Acinetobacter baumannii* complex, *Pseudomonas aeruginosa*, *Serratia fonticola* and *Hafnia alvei.* We were surprised by the relatively high rates of these potential pathogens and initially speculated that perhaps they were due to a point-source within certain supermarkets or butcher shops, such as has been reported in one outbreak of multidrug-resistant *K. pneumoniae* [35]. However, they were identified from both CD and PR products purchased from a wide variety of food outlets which had no common supply chain. Notably, only one *E. coli* isolate was identified – so testing programs which only assess for this species would have reported a much lower rate of potential contamination.

Secondly, our results highlight the importance of not relying solely on selective media such as ChromID ESBL agar in such programs, but instead confirming the presence of ESBL genes by PCR. Phenotypic detection methods alone may identify intrinsically 3GCR isolates or those that falsely suggest ESBL production [24–26]. AmpC genes were identified in 15% isolates assessed (17.5% specimens), with most (22/23) being inherently chromosomal in location [26]. Notably, however, the sole *E. coli* isolate identified contained a plasmid-mediated CMY-2 which was potentially transferable.

The fact that resistance to anti-folate agents was the most common resistance phenotype identified among potential ESBL-producing strains and was noted in 68.3% of all CD/PR specimens is important, given that trimethoprim-sulfamethoxazole is widely used in pork production and some chicken farms [15, 16]. Hence these results may be no surprise, but at least serve as a potential “wake up call” to farmers who are concerned about the consequences of frequent antibiotic use. Importantly, 9 isolates (7.5% CD/PR specimens) displayed an MDR phenotype, with only one strain (*Bordetella trematum*) being resistant to fluoroquinolones – consistent with Australia’s strict controls on fluoroquinolone use in agriculture and similar to previous studies on this issue [14].

*Acinetobacter*, *Serratia*, *Hafnia* and *Pseudomonas* spp. are all known to be common in the environment and to be present on some fruit and vegetables [36, 37], but their presence may be a potential source of resistance genes [38].

Given the uncertainty about which testing regimen would be ideally suited for a large national food safety screening program for MDR contamination [9–13], we believe our methodology was a practical approach that is potentially relevant and meaningful to retail consumers and which could be up-scaled without the need for major infrastructure or specialised training. In comparison, all previous published Australian studies have assessed non-meat items such as animal faeces or eggs [39–42].

Our findings differ from those by other authors. Overdevest et al. [8] reported that 79.8% of retail chicken meat samples in the Netherlands had organisms with ESBL genes present, while only 1.8% of pork samples grew an ESBL-producing organism. However, this study focused particularly on *E*. *coli* and *K*. *pneumoniae* without commenting on other organisms isolated. Stewardson et al. [7] reported 86% contamination of chicken meat products delivered to a tertiary hospital in Switzerland with ESBL-producing *Enterobacteriaceae* species. Similar to our results, MDR strains were uncommon.

This study has some limitations. Firstly, the sample size of 120 specimens, while consistent with similar studies, is relatively small in the context of overall Australian supply [2, 7, 8, 43–47]. Secondly, we were not able to track the original farm source of the CD and PR products, although one might expect larger supermarket chains to have a limited number of defined contracted suppliers. Further research to investigate the rates of contamination at each step of the meat production process, including samples from animals in farms, carcasses and meat products in slaughterhouses and of meat products distributed to third party organisations for packaging and distribution, may be helpful to identify if there is a common source of contamination. Thirdly, our sample preparation (including initial 24 h culture in non-selective media), the subsequent selective culturing techniques provided enhanced sensitivity for 3GCR-GNBs but did not allow us to accurately quantify the burden of contamination in each CD/PR sample. Importantly, we did not assess for phenotypic colistin resistance since laboratory methods are evolving [48, 49], nor did we assess for *mcr* genes since this resistance mechanism was only first reported in 2016 [50]. Notably, colistin resistance appears to be currently rare in Australia [51, 52] and colistin is infrequently used in Australian agriculture [2, 53]. Finally, Australia does not import fresh chicken meat, nor any fresh bone-containing pork products [13, 17], which means that all of our specimens came from animals born and grown in Australia. As such we cannot comment on any possible difference in contamination between these Australian products and similar, but boned, imported chicken and pork processed meat products.

We believe our findings raise important questions regarding future food testing programs and potentially highlight the importance of routine public health measures related to safe food preparation such as appropriate hand hygiene before/after handling uncooked meat products, adequate washing of kitchen utensils and surfaces that have contact with uncooked meat and appropriate cooking methods to ensure destruction of any contaminating bacteria. These public health messages may be of particular importance to patient groups where immunosuppression is likely, such as those with haematological malignancy or transplant recipients. Further research into the potential source(s) of retail meat contamination is warranted.

## Conclusion

Overall, we found low rates of MDR-GNBs in Australian chicken and pork meat, but potential 3GCR-GNBs are common (93% specimens), as is resistance to trimethoprim-sulfamethoxazole. Food testing programs that only assess for *E. coli* are likely to severely underestimate the diversity of 3GCR organisms in fresh meat.

## Additional file

Additional file 1: **Figure S1.** Location of 30 meat retailers in relation to Austin Hospital in eastern Melbourne. (DOCX 2275 kb)
