# Supplementary material for: Superbugs in the supermarket? Assessing the rate of contamination with third-generation cephalosporin-resistant gram-negative bacteria in fresh Australian pork and chicken
Source: Antimicrob Resist Infect Control. 2018 Feb 23;7:30. doi: 10.1186/s13756-018-0322-4 (PMC5824441; doi:10.1186/s13756-018-0322-4)

**Supplementary Figure 1.** Location of 30 meat retailers in relation to Austin Hospital in eastern Melbourne


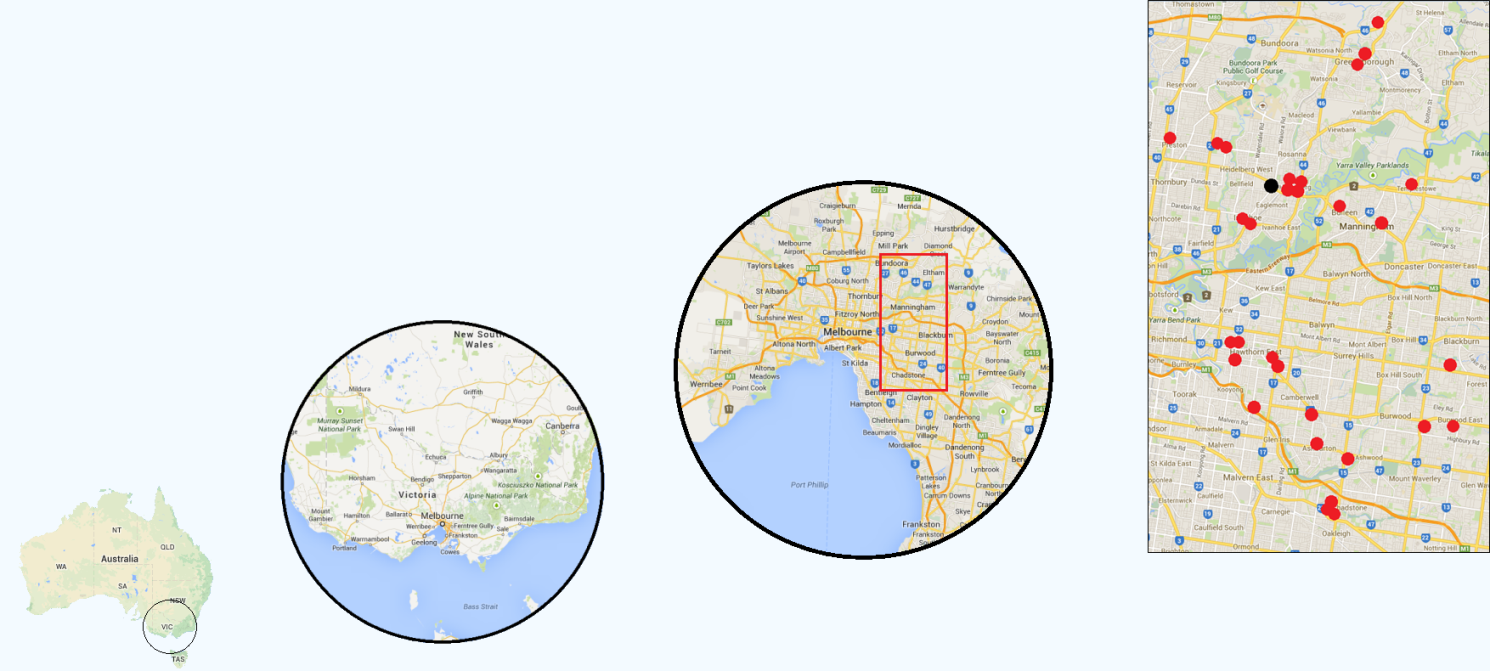

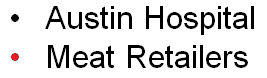

Supplement: Supplementary file 1 — Figure S1. Location of 30 meat retailers in relation to Austin Hospital in eastern Melbourne. (DOCX 2275 kb) [file 13756_2018_322_MOESM1_ESM.docx]
